# Theoretical and experimental study of the role of cell-cell dipole interaction in dielectrophoretic devices: application to polynomial electrodes

**DOI:** 10.1186/1475-925X-13-71

**Published:** 2014-06-05

**Authors:** Massimo Camarda, Giuseppe Fisicaro, Ruggero Anzalone, Silvia Scalese, Alessandra Alberti, Francesco La Via, Antonino La Magna, Andrea Ballo, Gianluca Giustolisi, Luigi Minafra, Francesco P Cammarata, Valentina Bravatà, Giusi I Forte, Giorgio Russo, Maria C Gilardi

**Affiliations:** 1 CNR-IMM Sezione di Catania, Z.I. VIII Strada 5, I-95121 Catania, Italy; 2 Dipartimento di Ingegneria Elettrica Elettronica e Informatica, Universita’ degli Studi di Catania, Catania, Italy; 3 Istituto di Bioimmagini e Fisiologia Molecolare (IBFM-CNR) - LATO, Cefalù, Sicilia, Italy

## Abstract

**Background:**

We aimed to investigate the effect of cell-cell dipole interactions in the equilibrium distributions in dielectrophoretic devices.

**Methods:**

We used a three dimensional coupled Monte Carlo-Poisson method to theoretically study the final distribution of a system of uncharged polarizable particles suspended in a static liquid medium under the action of an oscillating non-uniform electric field generated by polynomial electrodes. The simulated distributions have been compared with experimental ones observed in the case of MDA-MB-231 cells in the same operating conditions.

**Results:**

The real and simulated distributions are consistent. In both cases the cells distribution near the electrodes is dominated by cell-cell dipole interactions which generate long chains.

**Conclusions:**

The agreement between real and simulated cells’ distributions demonstrate the method’s reliability. The distribution are dominated by cell-cell dipole interactions even at low density regimes (10^5^ cell/ml). An improved estimate for the density threshold governing the interaction free regime is suggested.

## Background

As first defined by Pohl [1,2], the term “*dielectrophoresis*” is used to describe the *“ponderomotive”* force exerted by a non-uniform electric field on polarizable neutral particles. Such force allows for the controlled manipulation of micro and nano-sized particles dispersed in colloidal solutions. Application fields include: cell partitioning and isolation [3,4], bio-structure assembling [5], nanostructure (e.g. carbon nanotube) deposition [6], filtration systems for oils purification [7] etc. Among these, the separation of rare cells [8] is a specifically promising one as *dielectrophoresis* allows the capture/separations of cells without the use of biomarkers; relying, instead, in the strong selectivity of the dielectrophoretic (DEP) response [9] which depends on the particle mass, shape and composition. Indeed, recently, this selectivity has permitted to discriminate the tumor cell types of the NCI-60 panel from Peripheral Blood MonoNuclear cells (PBMNs) [10]. However, although many intriguing micro-structures have been fabricated in research laboratories, DEP devices have hardly gone beyond the proof-of-concept stage [11]. One of the problems that are hindering development and engineerization of the devices is the limited use of accurate numerical tools for their design which, in turn, is due to the computational complications arising by the particle-particle dipole interaction. Indeed, particle kinetics (i.e. the particle velocity field) in DEP devices can be easily calculated by mean of Poisson solvers and direct integration of the equation of motion only in the non-interacting particle approximation [12]. This approximation is not valid in the accumulation regions of the DEP devices where, due to the increased particle concentration, dipole-dipole interactions become important and can promote the formation of clusters and significant rearrangements of the particle space distribution [13-16]. These many-particle effects can be accurately simulated solving directly the equations of motion in the few-particles limit [17] i.e. this approach is not applicable for the simulation and design of realistic systems. Another possible approach is the use of reaction–diffusion models [18-20] but this approach needs an “ad hoc” parameter calibration to effectively consider the dipole-dipole interactions in compact models.

Recently a coupled Monte Carlo-Poisson (MC-P) method [21] has been implemented which allows simulating a large number of particles in large active zones (within the experimental range), explicitly including particle-particle interactions. The MC-P method has pointed out the relevance of this inclusion in the modeling predictions for the simplified condition of Two Dimensional (2D) electric field $\vec{E}\left(\vec{r}\right)$ distribution, where $\vec{E}\left(\vec{r}\right)$ explicitly depends on two space coordinates $\vec{E}\left(\vec{r}\right)\equiv \vec{E}\left(x,y\right)$ as in the case of very long interdigitated electrodes. However, the possibility to apply this approach for the numerical design of devices exploiting more complex fully Three Dimensional (3D) electric field distributions has not been yet demonstrated. Moreover the MC-P predictions have never been compared with real cell distributions in dielectrophoretic devices, in order to confirm their reliability. Aiming to the two objectives of the model extension and validation, we have improved the application potentiality of the MC-P method to simulate the features of devices generating 3D electric field distributions. In addition we applied the simulation method to the case of polynomial electrodes which are known to produce well defined 3D non-uniform electric fields and are used for the study of negative dielectrophoresis [22] or for the determination of particle dielectrophoretic response through electrorotation analysis [23,24]. We compare the simulated results with experimental distributions obtained in the same electrodes geometry to evaluate the role of p-p interactions and definitively demonstrate the predictive potential of this methodology.

## Methods

### Computational algorithm

A detailed description of the method can be found in ref. [21], here we summarize the key aspects of the simulations, specifically focusing on the 3D implementation.

In the first-order approximation of the polarization, an isolated particle immersed in a media and subjected to a non-uniform electric field $E\left(\vec{r}\right)=\vec{E}\left(\vec{r}\right)\exp\left(\mathrm{i\omega t}\right)$ oscillating at the *f* = *ω*/2*π* frequency, will be subjected to an effective averaged potential energy [25]:

(1) $${\bar{U}}_{\mathrm{eff}}\left(\vec{r}\right)=-\frac{1}{2}\alpha_{\mathrm{eff}}E_{\mathrm{rms}}^{2}\left(\vec{r}\right)$$

where $E_{\mathrm{rms}}\left(\vec{r}\right)=E_{\text{max}}\left(\vec{r}\right)/\sqrt{2}$ is the effective value of the varying electric field and *α*_*eff*_ is the average polarizability of the particle defined as:

(2) αeff=3VReϵ˜mReϵ˜1−ϵ˜mϵ˜1+2ϵ˜m=3VReϵ˜mRefCMω

where *V* is the particle volume, ${\tilde{\epsilon }}_{1,m}=\epsilon_{1,m}-i\sigma_{1,m}/\omega$ are the complex dielectric constants of the particle (1) and the media (m) and *f*_*CM*_(*ω*) is the Clausius-Mossotti factor which fully characterizes the dielectric response of the particle in the given medium. The isolated particle approximation holds only in the diluted density limit, i.e. only if the average distance between two particles in the colloidal solution is always very large otherwise an effective particle-particle interaction has to be considered as a result of the local distortion of the electric field lines generated by itself and by the other particles. In this case, the DEP force acting on each suspended particle can be directly calculated by means of the Maxwell tensor [9]: $\overset{↔}{T}\equiv T_{\mathrm{ij}}={\tilde{\epsilon }}_{m}\left(E_{i}^{\mathrm{tot}}E_{j}^{\mathrm{tot}}-0.5\delta_{\mathrm{ij}}E_{k}^{\mathrm{tot}}E_{k}^{\mathrm{tot}}\right)$ over the closing surface of the particles:

(3) $${\vec{F}}_{\mathrm{DEP}}=\oint \left(\overset{↔}{T}\cdot \vec{n}\right)\mathrm{dA}$$

Note that whereas the $\vec{E}$ field in Eq. 1 is the field generated by the external electrodes only, ${\vec{E}}^{\mathrm{tot}}$ in the Eq. 3 must be calculated *considering all the particle presence*. This direct calculation is not practically feasible in the kinetic simulation of large systems, since the particle distribution continuously changes in the space requiring an integration of the Poisson equation, $\nabla {\vec{E}}^{\mathrm{tot}}=0$, at each simulation step. A more efficient approach, that requires only the evaluation of the external field, can be implemented approximating the total distorted electric field with the sum of the field generated by the external electrodes plus the contributions of the dipoles induced in all the particles: $E^{\mathrm{tot}}\left({\vec{r}}_{i}\right)\approx E\left({\vec{r}}_{i}\right)+\sum_{j}^{\mathrm{all the particles}}E_{j}\left({\vec{r}}_{i}\right)$[26].

The reliability of this approximation has been demonstrated in Ref. [13] with the aid of the full calculation based on Eq. 3 for the case of two spherical particles immersed in a uniform external electric field: the magnitude, the angular dependency and the scaling with the distance of the calculated force are similar to those derived in the interacting dipoles approximation [26].

The effective potential energy that generalizes Eq. 1 for the case of particle-particle instantaneous interactions, in the dipole approximation when the multipole terms and the mutual polarization of the particles can be neglected, is:

(4) Uij≈−12Repir→i⋅Ejr→i*=−12Repjr→j⋅Eir→j*

where $\;\begin{array}{cc} E_{j}\left({\vec{r}}_{i}\right) & \left(E_{i}\left({\vec{r}}_{j}\right)\right) \end{array}$ is the electrical field generated by the dipole of the particle j (i) at the position $\begin{array}{cc} {\vec{r}}_{i} & \left({\vec{r}}_{j}\right) \end{array}$, while $\begin{array}{cc} p_{i}\left({\vec{r}}_{i}\right) & \left(p_{j}\left({\vec{r}}_{j}\right)\right) \end{array}$ is the dipole moment induced on the i-eme (j-eme) particle by the external field $\begin{array}{cc} E\left({\vec{r}}_{i}\right) & \left(E\left(r_{j}\right)\right) \end{array}$. The electric field in ${\vec{r}}_{i}$ generated by a particle in *r*_*j*_ is:

(5) E→jr→i=14πReϵm3n→n→⋅p→j−p→jRij3

where $\vec{n}={\vec{R}}_{\mathrm{ij}}/R_{\mathrm{ij}}$ which leads to the following expression for the average effective potential energy:

(6) U¯ij≅14πReϵmαeffiαeffj1−3cosθijicosθijjRij3E→rmsr→i⋅E→rmsr→j

where $\theta_{{}_{\mathrm{ij}}}^{i},\;\theta_{{}_{\mathrm{ij}}}^{j}$ are the angles between the vectors $\vec{E}\left({\vec{r}}_{i}\right),\;\vec{E}\left({\vec{r}}_{j}\right)$, and $\vec{n}$ and $\alpha_{\mathrm{eff}}^{i},\;\alpha_{\mathrm{eff}}^{j}$ are the average polarizations for the *i* and *j* particles.

We used a Monte Carlo methodology to efficiently determine the equilibration of a 3D system of interacting particles suspended in a static liquid medium under the action of an oscillating non-uniform electric field generated by polynomial electrodes. The particles are considered as hard spheres with radius *r*_*i*_ and the configuration energy is

(7) $$E\left(\left\{r_{1},\ldots r_{n}\right\}\right)=\sum_{i}U_{\mathrm{eff}}\left({\vec{r}}_{i}\right)+\sum_{i,j}{\bar{U}}_{\mathrm{ij}}\left({\vec{r}}_{i},{\vec{r}}_{j}\right)$$

where ${\bar{U}}_{\mathrm{eff}}$ and ${\bar{U}}_{\mathrm{ij}}$ are calculated by means of the equations (1) and (6).

The solution of the Poisson equation, which allows determining the 3D electric field spatial distribution, has been calculated by means of a finite element numerical solver [27]. Then, the numerical estimate of the electric field has been interpolated in the cubic grid of the MC simulation box and, in order to obtain an accurate resolution, the distance of next-neighbor nodes in the simulation grid has been set equal to the particle radius. Equilibration kinetics, which is reached by a sequence of stochastic jumps for the simulated particles, can be inferred from the simulation if Brownian motion and particle-particle scattering due to the hard-sphere behavior lead to an approximated diffusive behavior. In this case we can estimate the time interval *Δt* between two consecutive displacement events as

(8) $$N\Delta t≅1/6\times {\left(\mathrm{\Delta d}\right)}^{2}/\bar{D}\approx 1/6\times {\left(\mathrm{\Delta d}\right)}^{2}/D_{\mathrm{Brow}}=1/6\times {\left(\mathrm{\Delta d}\right)}^{2}\left(3\mathrm{\pi \eta a}\right)/k_{B}T$$

where $\bar{D}\approx D_{\mathrm{Brow}}$ is the effective diffusivity, *N* is the number of simulated particles, Δ*d* is the elementary particle displacement, *η* is the medium viscosity, *a* is the particle radius, *k*_*B*_ is the Boltzmann constant and *T* is the system temperature.

### Experimental setup

The polynomial electrode design described in the previous section, has been fabricated by deposition of 10 nm of Titanium followed by 200 nm of Nickel on a standard microscope glass. The electrodes were delineated by lithographic methods followed by wet etching. The device has been energized using a Protek 9205C signal generator which applied, consistently with the simulated systems, a sinusoidal voltage signal of 8V_pp_ value at 1 MHz for 180 sec (long time allow for cells equilibration). The final distribution was observed with a standard 10× phase contrast inverted microscope. The human breast cancer cell line MDA-MB-231 were cultured according to American Type Culture Collection (ATCC) instructions. The cells, just before DEP tests, were suspended in a low conductive buffer (used as the elute in all our experiments) composed of 9.5% ultrapure sucrose (S7903, Sigma-Aldrich), 0.3% dextrose (Fisher D-16), and 0.1% Pluronic F68 (P1300, Sigma-Aldrich) titrated to a conductivity of 30 mS/m (consistent with Monte Carlo simulations) by adding KCl with the aid of a conductivity meter. The buffer had an osmolarity of 320 mOs/L and a pH of 7 and the experiments were conducted at room temperature (22°C). The cells, suspended in DEP buffer at concentration of 5×10^5^ cells/ml were pipetted into the chamber and occupied a total volume of about 100 μl when a cover slip was placed over the rubber o-ring.

## Results

**Figure 1:**
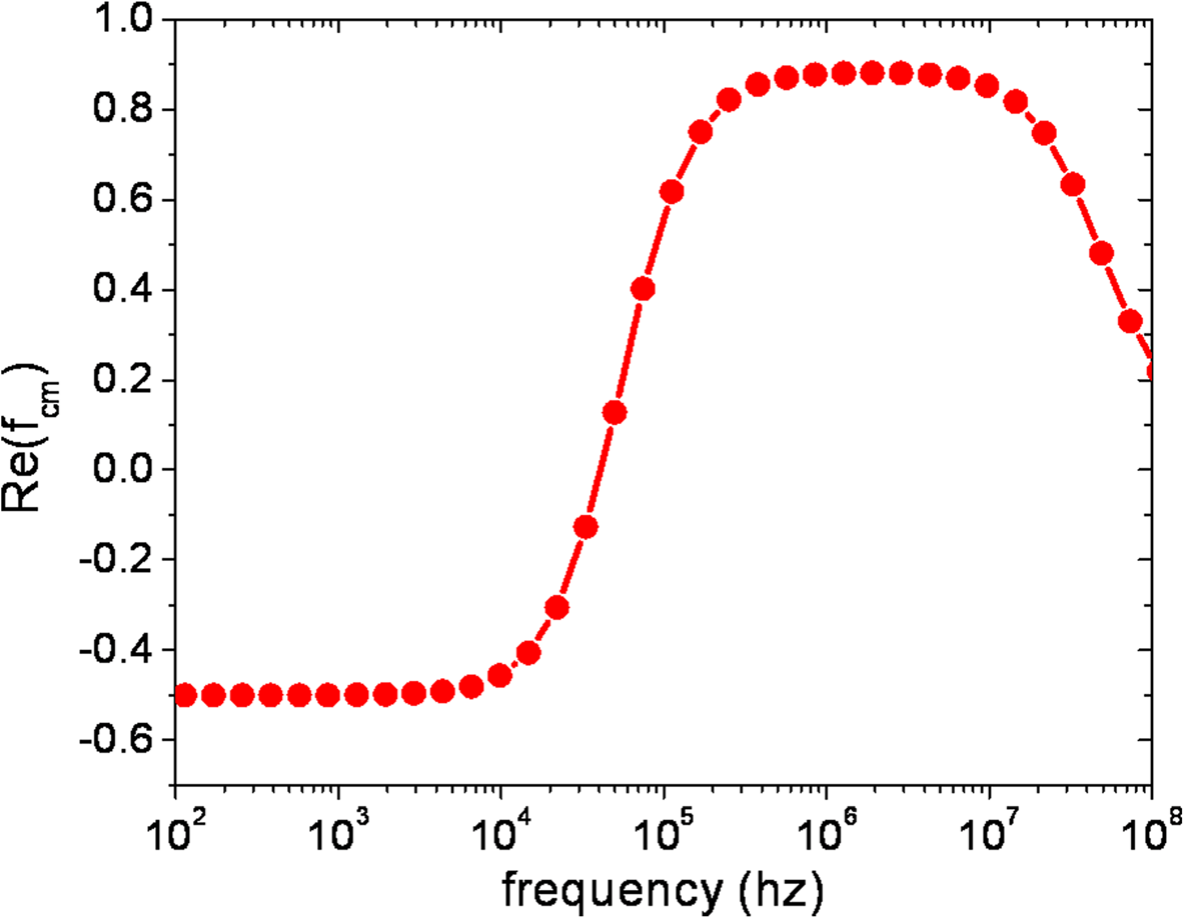
**Real part of the Clausius-Mosotti factor *f***_***CM***_**(*ω*) calculated with the dielectric model of the MDA-MB-231 cell at a medium conductivity of 30 mS/m.** At the frequency of *f =* ω*/2*π = 10^6^ Hz, used for both simulations and experiments, the cells are subjected to a positive-DEP, i.e. they will tend to move towards high electric field regions.

In order to study the interplay between the dielectrophoresis response and the particle-particle dipole interaction in a realistic case, we consider a suitable dielectric model of a colloidal system of MDA-MB-231 cells dispersed in a weakly conducting water solution (see later). The complex dielectric constant ${\tilde{\epsilon }}_{\mathrm{eff}}$ of these cells can be approximated by the solution of the following system of equations:

(9) $$\begin{array}{c} {\tilde{\epsilon }}_{\mathrm{eff}}=\frac{1+2dr^{3}A}{1-dr^{3}A}\\ \mathrm{dr}=\frac{a}{a+d}A=\frac{{\tilde{\epsilon }}_{\mathrm{in}}-{\tilde{\epsilon }}_{\mathrm{me}}}{{\tilde{\epsilon }}_{\mathrm{in}}+2{\tilde{\epsilon }}_{\mathrm{me}}} \end{array}$$

where ${\tilde{\epsilon }}_{\mathrm{in},\mathrm{me}}=\epsilon_{\mathrm{in},\mathrm{me}}-i\sigma_{\mathrm{in},\mathrm{me}}/\omega$ are the inner cell and membrane complex dielectric constants and *a = 6.2* μ*m* and *d = 10 nm* are the cell radius and membrane thickness**.** The following values have been used: $\epsilon_{\mathrm{me}}^{'}=24\epsilon_{0},\;\epsilon_{\mathrm{in}}^{'}=50\epsilon_{0},\;\sigma_{m}=30\;\mathrm{mS}/m,\;\sigma_{\mathrm{in}}=0.2\;S/m,\;\sigma_{\mathrm{me}}=10^{-7}S/m$, [28], and the calculated real part of the factor *f*_*CM*_(*ω*) is shown in Figure 1.

**Figure 2:**
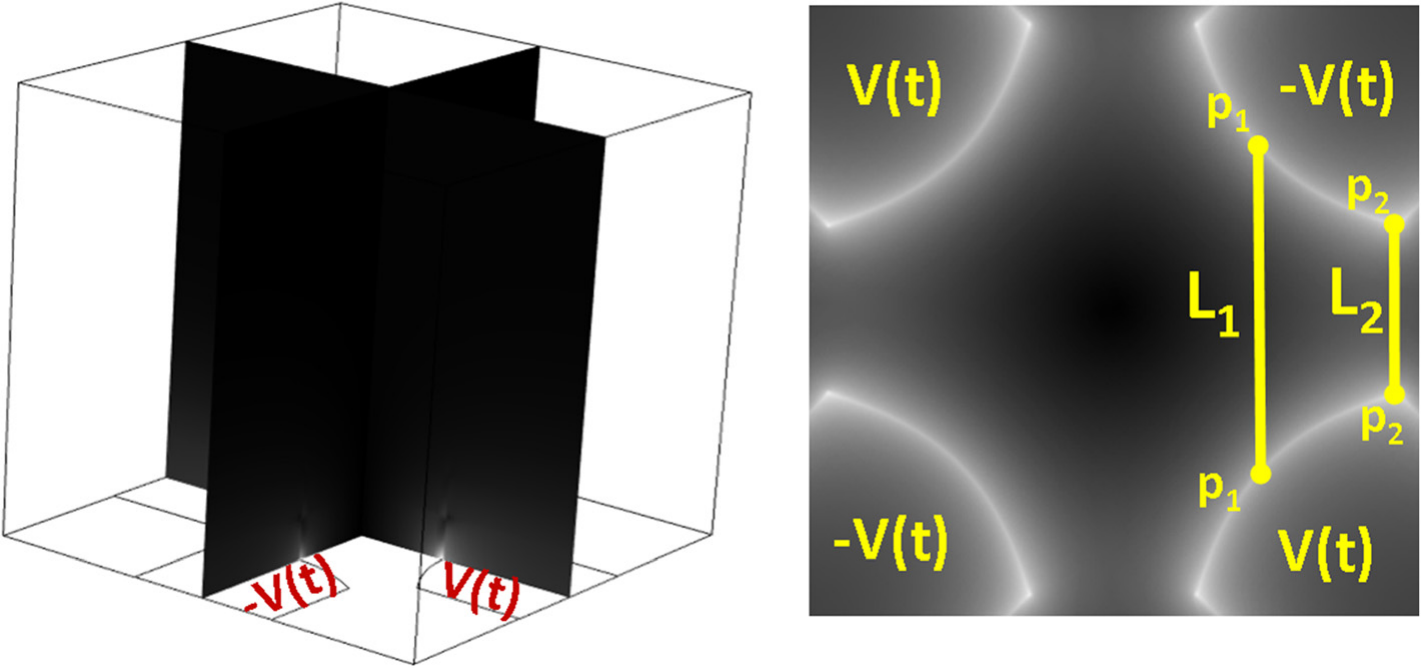
***Left,*side view of the simulated computational box (1600×1600×1500 μm**^**3**^**) with, superimpose in grayscale, intensity map of **$\left|E_{\mathrm{rms}}\right|\left(\vec{r}\right)$**.***Right*, Top view of the system, the intensity map of $\left|E_{\mathrm{rms}}\right|\left(\vec{r}\right)$ shows the areas of higher electric field (brighter areas). *L*_*1*_ and *L*_*2*_ are the distances between the opposing electrodes in *p*_*1*_ and *p*_*2*_, respectively.

The cells are free to move in a cubic computational box of dimensions 1600×1600×1500 μm^3^ with four polynomial electrodes located at the bottom of the box (see Figure 2) whose shapes can be described by the following parametric system:

(10) $$\begin{array}{c} D\leq x\leq L\\ y=\pm \sqrt{x^{2}+D^{2}} \end{array}$$

for each electrode [22]. *D* represents half the distance of opposing electrodes whereas *L* is related to the electrode width. Referring to Figure 2, *right* and using Eq. 10, we have that $L_{1}=\sqrt{2}D$ and $L_{2}=\sqrt{2}\left(L-\sqrt{L^{2}-D^{2}}\right)$. To improve the capturing efficiency of the DEP system the electric fields in *p*_*1*_ and *p*_*2*_ must be of the same order (otherwise the trapping regions will be limited to the *p*_*2*_ regions). Given that |*E*_*rms*_|(*p*_1_) ∝ *V*_*pp*_/*L*_1_ and |*E*_*rms*_|(*p*_2_) ∝ *V*_*pp*_/*L*_2_*D* must be of the same order of *L*, in this study we sat *D* = 390 μm and *L* = 460 μm (note that in ref. [22]*D =* 64 μm and *L* = 90 μm which favored negative-DEP only). Figure 2, *left* shows the simulated box with the four electrodes situated at the bottom. The intensity map of $\left|E_{\mathrm{rms}}\right|\left(\vec{r}\right)$ is also reported in grey scale for the oscillating four-electrodes configuration at a frequency of 1 MHz and a peak value of 8 V for *V*_*pp*_ with 180° phase difference between neighbor electrodes (i.e. *V*_1_(*t*) = − *V*_2_(*t*) = *V*_*pp*_*sin*(2*πft*)). Figure 2, *right* shows the electric field at 50 μm from the bottom surface, associated to the considered system. The regions of high electric field (to which the cells will move under positive-DEP) are located all around the edges of the electrodes. In the considered system the highest electrical fields are located at *p*_*1*_ and *p*_*2*_ equivalent points.

**Figure 3:**
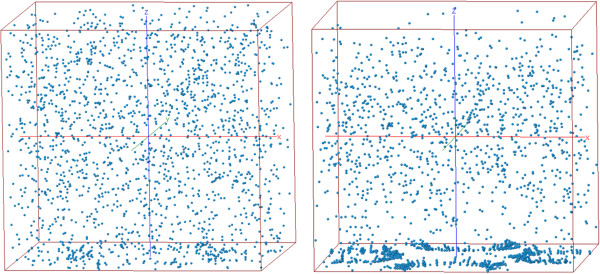
**Initial (*left*) and final (*right*) spatial distribution of N = 1920 MDA-MB-231 cells suspended in a saline water solution of conductivity of 30mS/m subjected to an oscillating electric field *f =*** **10**^**6**^ **Hz.** The cell distribution is obtained starting from a random distribution after *2*×*10*^*8*^ MC iterations. Dipole chains are evident at the bottom of the simulation box and are associated to high regions of electric field.

In Figure 3 we show the initial random distribution of 1920 cells (corresponding to a density of 5×10^5^ cells/ml) (*left*) and the final (*right*) equilibrium condition after *2*×*10*^*8*^ Monte Carlo iterations. The final distribution is the result of the minimization of Eq. 7, which induce a movement towards the *p*_*1*_ and *p*_*2*_ regions (minimization of the first term) and an aligned of the cells (causing cell-chains) along the electric field direction (minimization of the second term). Specifically particles tend to form larger chains in the regions of more intense electric field which are located mainly around *p*_*2*_ equivalent points, similar distributions have been observed for densities as low as 1×10^5^ cells/ml (not shown). It is important to note that this tendency does not depends on the DEP polarity because the $\alpha_{\mathrm{eff}}^{i}\alpha_{\mathrm{eff}}^{j}$ term in Eq. 7 is always positive for identical cells (whereas the $\alpha_{\mathrm{eff}}^{i}$ term in Eq. 1 can be positive or negative, depending on the value of *f*_*CM*_(*ω*)).

From these results we can infer that particle-particle interactions compete with the dielectrophoretic force-field, which would otherwise massively trap (in p-DEP conditions) the particles in the regions where the gradient of the electric field is larger. Note also the cells in the region far away from the electrodes which are not trapped by the DEP field, this allows for the definition of a depletion volume as the region where cells are effectively attracted to the electrodes. The connection between depletion region, electrodes geometry and particle-particle interaction is currently under investigation.

**Figure 4:**
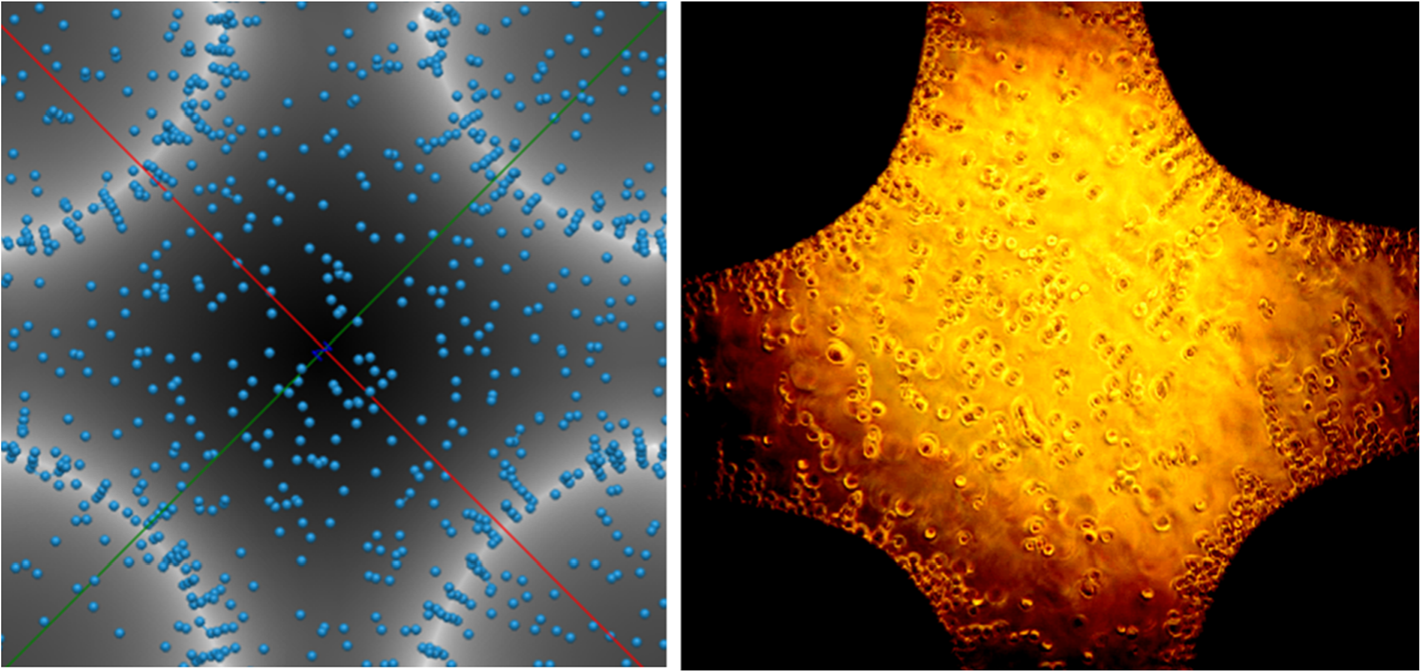
***left*) Top view of the final spatial distribution of the Monte Carlo simulation (same of Figure** 2**) with superimposed the intensity map of **$\left|E_{\mathrm{rms}}\right|\left(\vec{r}\right)$**(same as Figure** 2**,*right*).** The association between dipole chains and high regions of electric field is evident. *Right*) Top view of experimental spatial distribution of MDA-MB-231 after 180 sec of DEP manipulation. Black areas are associated to the metal electrodes. The chains are clearly present all around the electrodes.

Figure 4 shows a comparison between the simulation and the experimental cells distribution. As can be seen the two distributions are equivalent when the statistical approach of the equilibration is considered. In both cases the cells distribution near the electrodes is dominated by cell-cell dipole interactions which generate long chains. Note also the, relative small, discrepancy between the simulated and experimental cell distribution in the region at the center of the four electrodes. One possible explanation of this weak discrepancy is the current preliminary calibration of the friction forces between the cells and the wall on the bottom of the chamber (this force is included in the MC code). Work is underway to improve the parameter calibration and definitively refine the simulation results. The highest value of the cell concentration to avoid particle-particle interactions strongly depends on the electrodes and system geometry, on the particles dimensions and on the polarization factors. As a consequence a general prescription to neglect dipole-dipole interaction in the design of a device cannot be easily given. Jones [29] showed that cell-cell interactions should become significant when the ratio of cell spacing δ to cell diameter *d* falls below ~5. In a simple approximation we could assume that all cells will eventually settle to the bottom of the chamber, which gives:

(11) $$\delta /d=1/\left(dh^{1/2}C^{1/2}\right)>5\to C<C_{\mathrm{threashold}}=\frac{4\times 10^{8}}{hd^{2}}\mathrm{cells}/\mathrm{ml}$$

where *h* is the chamber height and *C* is the cell concentration. Setting *h* = 1500 μm and *d* = 2 × *r* = 12.4*μm* we obtain *C* < 5 × 10^5^cells/ml. According to this rough estimate, the density used in the Monte Carlo simulation (*C* = 10^5^ cells/ml) should not lead to significant dipole-dipole interactions. The limit of this simple description is that it implicitly assumes a uniform distribution of cells at the bottom of the chamber, i.e. it neglects the fact that the cells, due to the DEP field, will concentrate at the electrodes edges (see Figure 4). In order to qualitatively take it into account this effect, it is possible to correct the formula using the following parameter:

(12) $$r_{\mathrm{DEP}}={\left(\nabla E^{2}\right|}_{\mathrm{Max}}/\langle \nabla E^{2}\rangle$$

where ∇*E*^2^|_*Max*_ and 〈∇*E*^2^〉 are, the highest and average value of the electric field gradient calculated at the bottom of the chamber. Since the dielectrophoretic force is proportional to the electric field gradient, *r*_*DEP*_ approximately represents the concentration factor generated by the DEP force. So that we improve the previous Eq. 11 as:

(13) $$C_{\mathrm{threashold}}=4\times 10^{8}\frac{1}{hd^{2}r_{\mathrm{DEP}}}\mathrm{cells}/\mathrm{ml}$$

i.e. the highest the concentration ratio, the lower the threshold will be. Another assumption of Eq. 11 is that *all* the cells settle at the bottom of the chamber. This is not generally true (see Figure 3) and it depends on the allowed deposition time and on the electric fields. To take this into account we need to substitute, in Eq. 13 the chamber height *h* with the effective capturing region (*z*_*capture*_):

(14) $$\begin{array}{c} C_{\mathrm{threashold}}=4\times 10^{8}\frac{1}{z_{\mathrm{capture}}d^{2}r_{\mathrm{DEP}}}\mathrm{cells}/\mathrm{ml}\\ \int_{0}^{z_{\mathrm{capture}}}\langle v_{z}\left(z\right)\rangle \mathrm{dz}=\int_{0}^{z_{\mathrm{capture}}}\frac{\langle \nabla_{z}U_{\mathrm{eff}}\left(z\right)\rangle }{6\pi \mu_{m}r_{\mathrm{cell}}}\mathrm{dz}=t_{\mathrm{dep}} \end{array}$$

Where 〈∇_*z*_*U*_*eff*_(*z*)〉 is the average DEP force in the vertical direction at distance z from the bottom of the chamber and μ_*m*_ is medium viscosity. In the case of the polynomial electrodes used and for a deposition time of 180 sec, *r*_*DEP*_  ≅ 85 and *z*_*cap*_ = 280*μm* ≅ (1/5)*h* so that the improved concentration threshold, to avoid dipole-dipole interaction, should be below 3 × 10^4^ cells/ml. We performed Monte Carlo simulations in the 10^4^ cells/ml range finding no significant evidence of cell chains formation (not shown), thus confirming that Eq. 14, together with Eq. 12, represent a better qualitative threshold to avoid cell-cell interaction requiring only a knowledge of the electric field in the DEP device.

## Conclusions

In conclusion, we have demonstrated that the effects of particle-particle interactions play a crucial role in the kinetic evolution of colloidal systems in DEP devices even at low density regimes (10^5^ cells/ml), lower than the ones currently used in DEP devices [30].

Monte Carlo methods allow for the simulation of sufficiently large systems in terms of size and number of particles (i.e. within the experimental scopes). The discrete approach (i.e. the particle resolution), as opposed to the fluid-flow methodologies, is the key ingredient of the method improvement. In the case of MDA-MB-231 tumor cells suspended in a static, low conductive, fluid under the action of a positive-DEP field generated by a polynomial schema we have elucidated the crucial role of particle-particle interactions on the trapping efficiency of the device, on the organization of cells in ordered chains and on the overall cell space distribution. We have also deduced a new qualitative concentration threshold to avoid cell-cell interaction which requires only a knowledge of the electric field in the DEP device. Clearly, to have an exact determination of the concentration threshold for the specific DEP device used, MC-P kinetic simulations varying the cells density, such as the one proposed in this paper, need to be performed.

Future works will be devoted to generalize the formalism here presented in order to include second-order effects such as cell-sedimentation and cell-stitching or including hydrodynamic forces to simulate cells distributions in dynamic separation systems.

## Competing interests

The authors declare that they have no competing interests.

## Authors’ contributions

MC, GF, ALM have contributed to the computational algorithm. RA, SS, AA and FLV have contributed to the realization of the dielectrophoretic devices. AB and GG have designed the electronic components. LM, FC, VB, GIF, GR and MCG contributed to the biological part, culturing and handling the MDA-MB-231 cells. ALM is the coordinator of the research work. All authors conceived the study, interpreted the data and drafted the manuscript. All authors read and gave final approval for the version submitted for publication.
